# Knowledge on the Minamata Convention on Mercury and dental amalgam phase down, availability and selection of mercury-free restorative materials among dentists in Kenya: a cross-sectional study

**DOI:** 10.11604/pamj.2025.52.1.44453

**Published:** 2025-09-01

**Authors:** Bernina Kyale Kisumbi, Loice Warware Gathece, Olivia Awino Osiro, Susan Wanjiku Maina

**Affiliations:** 1 Unit of Conservative and Prosthetic Dentistry, Department of Dental Sciences, University of Nairobi, Nairobi, Kenya,; 2 Unit of Periodontology/Community and Prosthetic Dentistry, Department of Dental Sciences, University of Nairobi, Nairobi, Kenya

**Keywords:** Dental amalgam, dental caries, mercury, dentists, composite resins, glass ionomer cements

## Abstract

**Introduction:**

the Minamata Convention on Mercury has dawned a paradigm shift from the use of dental amalgam to a dental caries preventative, minimally invasive dentistry model that employs mercury-free alternatives. The aim was to assess Kenyan dentists’ knowledge on the Minamata Convention on mercury (MCM) and dental amalgam phase down (DAPD), availability, and selection of amalgam alternative restoratives.

**Methods:**

the study targeted n=260 dentists in November 2022. Data was collected online using an anonymous semi-structured questionnaire which was developed by the researcher, reviewed by a dental faculty experts panel, and received input from a pilot study. Descriptive statistics were used for data analysis.

**Results:**

among the 256 participants, the majority knew the reason behind DAPD (61.3%), and what is crucial for countries in its implementation (68%). Few 39.1% were knowledgeable on Kenya’s position concerning MCM as of 2022. The majority (71.3%) used composite resins in permanent posterior teeth. In restoring very large carious lesions in permanent teeth, 68.4% selected composite resins often and always. Conventional glass ionomer cements were the most (68%) available alternative restorative. Most (87.7%) prepared cavities to manage early caries lesions at the enamel dentinal junction, while 50.3% would be restored with composite resins.

**Conclusion:**

Kenyan dentists were knowledgeable on the MCM and DAPD. The majority selected composite resins to restore very large cavities while the most accessible alternative was conventional glass ionomer cement. DAPD is advanced in Kenya; caries prevention and wider availability of the stronger restoratives should be enhanced.

## Introduction

The dental amalgam phase-down (DAPD), enshrined in the Minamata Convention on Mercury, has increased patients´ demand for tooth-colored restorations. This has catalyzed a decrease in the use of dental amalgam in favour of alternative restorative materials in both clinical practice and teaching globally [1-3]. This has dawned a paradigm shift from the “*drill and fill*” dental amalgam (DA) restorative model towards a dental caries preventive and minimally invasive dentistry approach [4,5]. Dentists will be required to be knowledgeable on the DAPD and the impact of a sudden ban on dental amalgam to participate in the formulation of the national phase-down policies to avert the widening of oral health inequalities [3]. A recent study among final year dental students and interns in Kenya showed a high level of knowledge on the MCM and competency in using dental amalgam alternatives [6]; however, the situation among the dentists in Kenya is not yet established.

A study on the knowledge of MCM and DAPD among Jordanian dentists found that this did not impact the reduction in use of DA [2]; however, it informed the dentists are changing contemporary restorative models and may have influenced their selection of suitable dental amalgam alternative restorative (DAARs) and execution of quality restorations. With the varied circumstances of the phase-down recorded globally, from the phase-out status in some countries to continued use of DA as the mainstay restorative in others [3,7-10], the availability of the alternative restoratives and appropriate skills for correct selection and indication remains imperative. The situation is critical in low- and middle-income countries where oral health is not prioritized and the gap in public and private practice complicates access to quality care [11,12]. A study done in 2009 in Kenya reported preference of DA for posterior fillings, and only one study in 2014 singled out the application of DAARs in posterior restorations, reporting low usage [13], but the situation a decade post the MCM is largely unknown.

All DAARs have been available in Kenya, as reported in a 2016 study [13], but the level of access dentists across the country have not been investigated. Moreover, concerning very large cavities in molars and premolars that were previously built up using DA, there is scanty information on the uptake of alternative indirect materials that include composite resins, gold alloy, and ceramics, and for inlays and onlays in Kenya. There is scientific evidence that indirect composite resins, including short fiber resin composites, are suitable DAARs for large restorations in socioeconomic situations. Very large direct DAAR restorations of the most commonly used DAAR composite resins have been associated with the occurrence of bulk fractures and endodontic treatment failures [14,15]. Kenya became a Party to the MCM in late 2023; therefore, baseline data will be required to inform the development of national DAPD guidelines and subsequently policy to guide the process.

**Objectives:** the broad objective was to assess Kenyan dentists´ knowledge of the MCM and DAPD and the selection and availability of mercury-free alternative restoratives nearly a decade after the Minamata Convention on Mercury came into force. While the specific objectives were to assess the dentists´ level of knowledge on the MCM and DAPD, to evaluate the skill in selection of alternative restoratives for very large and early carious lesions, and to explore the availability of alternative restoratives in their facilities.

## Methods

**Study design and setting:** an online descriptive cross-sectional study was done among dentists in the 47 counties. Kenya is an East African country that serves as the major economic center in the region. The country has an area of 80,367 sq. kms, and was recently divided into 47 semi-autonomous counties. Health services are managed at the devolved level, hence dentists often encounter varied practice environments. Nevertheless, online platforms somewhat unify the country´s oral health primary care providers to share and access information.

**Study participants:** the study included intern dentists, general dentists, and specialists who were registered by the Kenya Medical and Dental Practitioners Council in November 2022. There were 742 dentists who were licensed to practice and whose contacts were retrievable. The inclusion criteria were dentists who consented to the study, while the exclusion criteria were dentists who did not execute restorative procedures and did not plan to do so after the training.

**Study size:** sample size was calculated using the Yamane Taro formula for a finite study population [16]. For a population of 742:


$$n=N/1+Ne^{2}$$

Where n is the minimum sample size, N is the study population, and e is the 5% margin of error. Substituting all a sample size of 260 was targeted.

**Sampling frame and technique:** purposing sampling was done via a bulk short text message that was sent to all dentists using the national dental association directory. It included a *Limesurvey link* that invited dentists to participate in the survey and submit their telephone numbers. Subsequently, a study *WhatsApp* wall was formed, which served as the communication channel. There were 320 who submitted their telephone numbers and 314 who remained in the *WhatsApp* wall.

**Data collection:** data were collected using an anonymous semi-structured questionnaire, which was developed by a panel of four dental faculty members, all of whom were experts in the subject of study and research. Sociodemographic characteristics garnered included: sex, age, highest qualification, country of obtaining basic degree, year of obtaining primary dental qualification, sector of practice, county work station, and practice of restorative dentistry. To assess knowledge of MCM and DAPD, four multiple-choice questions (MCQs) were used. Each question had four statements, totaling 16 options from which participants were to choose the single best answer. Following this, frequencies were computed to show how many participants answered correctly and incorrectly. The MCQ questions are provided in the results section (Table 1). The checklist consisted of nine alternative restoratives to which the participants were to indicate if they were available in their facilities or not.

**Table 1:** frequency distribution of Kenyan dentists (n = 256) based on their knowledge of the Minamata Convention on Mercury and the dental amalgam phase-down, as of 2022

Knowledge area/MCQ question	Knowledgeable; n (%)	Not knowledgeable; n (%)
Kenya's position pertains to the MCM	100 (39.1)	156 (60.9)
What is the major reason why dental amalgam is being phased down?	157 (61.3)	99 (38.7)
What is considered important as countries globally phase down the use of dental amalgam?	174 (68)	82 (32)
Among the DAARs for posterior restorations in permanent premolars and molars, which of the following is the most commonly applied?	214 (84)	42 (16)

MCM: Minamata Convention on mercury; DAARs: dental amalgam alternative restoratives

The section on the selection of restorative materials was based on a questionnaire by Espelid and Tveit [17], which has been used in several previous studies globally. It included sketches of proximal early carious lesions to which the participants were asked to indicate their management of choice. The various restorative materials were listed, to which the participants were to indicate the level of usage “Never”, “Rarely”, “Often”, “Sometimes”, and “Always”. The section on the selection of intervention and restorative material for early carious lesions included an open-ended question. Participants were to select the intervention and respective restorative criteria for each of the levels of early carious lesions. To ensure validity and consistency, the questionnaire was piloted among 26 dentists, and input was used to improve the tool. The outcome variables were the level of knowledge on MCM and DAPD, skill in selection of restorative, and the level of availability of dental amalgam alternative restoratives. A link to the questionnaire was sent to participants in the study *WhatsApp* wall who filled and submitted their responses online via the *Limesurvey tool (LST)* on 15^th^ November 2022. To minimize bias, the inclusion and exclusion criteria were adhered to, questionnaires were anonymous, and the participants were asked to answer all questions; this was in an attempt to avoid missing data.

**Statistical analysis:** data was exported from *Limesurvey* to Statistical Package for the Social Sciences version 25, *IBM Chicago, Illinois* which was used for analysis. Descriptive statistics, namely frequencies, percentages, and standard deviation, were computed to analyze data on sociodemographic characteristics, selection of restorative materials for the cavities presented availability of DAARs variables.

**Ethical considerations:** ethical approval was sought from the Kenyatta National Hospital/University of Nairobi Ethics, Research and Standards committee, approval number P616/07/2021, and a research permit was obtained from the National Commission for Science, Technology and Innovation (NACOSTI), license no. NACOSTI/P/22/17072. Consent to undertake the study was obtained from the respondents in the first part of the online questionnaire, which provided a brief explanation of the study. The data collected were anonymous, and participants were informed of their right to withdraw from the study at any time.

## Results

**Sociodemographic characteristics:** out of the targeted sample size of 260, 256 filled the questionnaire, a response rate of 98.8%. Dentists from 28 out of the 47 counties in Kenya participated. Almost half (43.4%) of the participants worked in four counties that house the four largest cities, whereas the remainder were from 24 counties. There were more males (59.8%), while the mean age of the dentists was 37.6 (SD 10.26), and a minority (20.6%) were over 50 years. Almost all the dentists (95.3%) practiced restorative dentistry, with 68.7% having obtained their basic degree after the year 2000 (Table 2).

**Table 2:** frequency distribution of Kenyan dentists based on their knowledge of the Minamata Convention on Mercury and dental amalgam phase-down among participants (n = 256) as of 2022

Categories	Dentists; n (%)
**Sex**	
Male	153 (59.8)
Female	103 (40.2)
**Age in years**	
<30	63 (24.0)
30-34	53 (20.7)
35-39	53 (20.7)
40-44	25 (9.4)
45-49	12 (4.6)
≥50	50 (20.6)
**Highest qualification**	
BDS	166 (69.2)
Masters	73 (30.4)
PhD	1 (0.4)
Other	16 (6.3)
**Country of obtaining a basic degree**	
Kenya	229 (88.7)
Other	27 (11.3)
**Year of primary qualification**	
<1960	14 (5.5)
1960–1980	21 (8.2)
1980–2000	45 (17.6)
2000>	176 (68.7)
**Sector of practice**	
Public	39 (15.2)
Private	107 (41.8)
Both public and private	100 (39.1)
Faith based	10 (3.9)
**County of work station**	
Counties with large Cities*	111 (43.4)
Other	145 (56.6)
**Practicing restorative dentistry**	
Yes	244 (95.3)
No	12 (4.7)

* Nairobi, Mombasa, Kisumu, and Nakuru; BDS: bachelor of dental surgery; PhD: doctor of philosophy

**Knowledge on the Minamata Convention on Mercury and dental amalgam phase down:** the participants were knowledgeable about three out of the four areas of MCM and DAPD that were evaluated. A sizeable number (84%) indicated correctly that among the DAARs, composite resins are the most commonly used for posterior permanent teeth. While a majority knew the reason behind DAPD (61.3%), and what is crucial for countries in its implementation (68%). However, only 39.1% were knowledgeable on the status of Kenya with regard to the MCM as of 2022 (Table 1).

**Restorative dental materials to restore a very large mesio-occlusal distal cavity:** a majority of dentists, 68.4% would select composite resins to restore a very large mesio-occlusal lesion (>2/3 intercuspal width) in permanent molars and premolars “often” and “always”. While the second and third restorative materials that would be selected by the dentists “often” and “always” were dental amalgam and resin-modified glass ionomer cements, 36.7% and 25.8% respectively. A minority of the dentists, 14.1% would select ceramic inlays “often” and “always”. On the other hand, a sizeable number of dentists stated that they would never select alkasite restorative material, 64.1% and gold inlays, 52.0%. Notably, there was a minority who never selected dental amalgam, 23.4% (Table 3).

**Table 3:** frequency distribution of Kenyan dentists (n = 256) based on their selection of restorative dental materials for managing very large mesio-occluso-distal (MOD) cavities (>2/3 intercuspal width) as of 2022

Restorative material	Never; n (%)	Rarely; n (%)	Sometimes; n (%)	Often; n (%)	Always; n (%)
Composite resins	7 (2.7)	6 (2.3)	68 (26.6)	98 (38.3)	77 (30.1)
Dental amalgam	60 (23.4)	46 (18.0)	56 (21.9)	45 (17.6)	49 (19.1)
Conventional glass ionomer cements	77 (30.1)	77 (30.1)	52 (20.3)	14 (5.5)	36 (14.1)
Resin-modified glass ionomer cements	59 (23.0)	51 (19.9)	80 (31.3)	30 (11.7)	36 (14.1)
Glass hybrid	105 (41.0)	44 (17.2)	50 (19.5)	15 (5.9)	42 (16.4)
Compomer	110 (43.0)	53 (20.7)	40 (15.6)	14 (5.5)	39 (15.2)
Alkasite restorative material	164 (64.0)	28 (10.9)	14 (5.5)	5 (2.0)	45 17.6
Ceramics	90 (35.1)	69 (27.0)	61 (23.8)	20 (7.8)	16 (6.3)
Gold alloy	133 (51.9)	43 (16.8)	26 (10.2)	12 (4.7)	42 (16.4)

**Figure 1:**
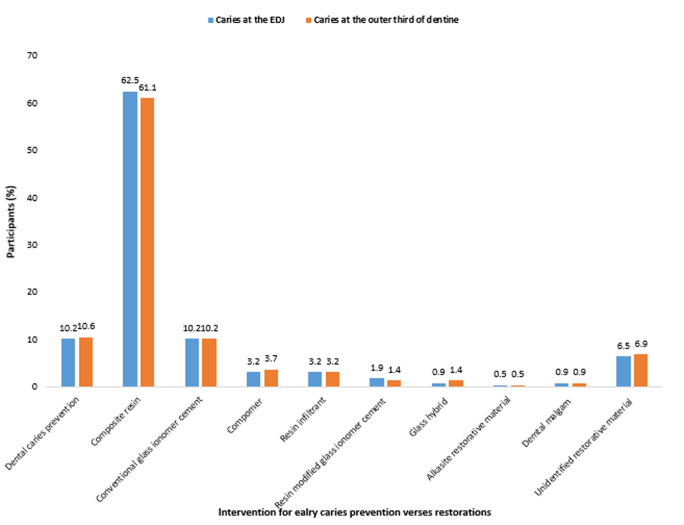
frequency distribution of Kenyan dentists (n = 256) according to their chosen intervention for early caries preventive measures versus restoration as of 2022

**Management of early dental caries:** almost all participants (87.7%) reported they would restore caries at the enamel-dentine junction (EDJ), while 98.6% indicated they would restore caries extending to the outer third of the dentine. The most preferred restorative material for management of dental caries at the EDJ and outer third of dentine was composite resin, 62.5% and 61.1% respectively, followed by an equal number, 10.2% that would use conventional glass ionomer cements. For both caries extents, less than 1% of the dentists would select glass hybrid, alkasite restorative material, and dental amalgam. In contrast, only a minority of the dentists, 10.2% would execute preventive intervention for caries extending to EDJ, and 10.6% for caries extending to the outer third of dentine (Figure 1).

**Figure 2:**
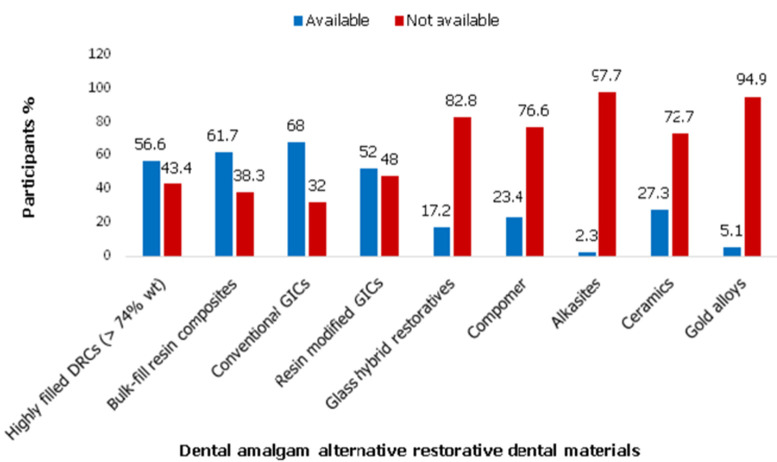
frequency distribution of Kenyan dentists (n = 256) according to the reported availability of alternative restorative materials to dental amalgam in their facilities as of 2022

**Availability of dental amalgam alternative restoratives:** among the nine DAARs, four direct restoratives were at the disposal to more than 50% of the dentists. Conventional glass ionomer cement was available to the majority of participants (68.0%), followed closely by bulk-fill resin composites (61.7%). Among the listed restorative dental materials, the least available were alkasite restorative materials (2.3%) and gold alloys 5.1% (Figure 2).

## Discussion

The response rate was high, which may be explained by the fact that DAPD has been a global topical issue in the past decade. It was higher than that of studies among dentists: 2.7% of 14,890 in Australia, 5.2% of 45,557 in the USA, and 45% of 1686 in Jordan [2,18,19]. It falls between the range reported in a recent systematic review on online dental surveys, 7% - 100%, mean of 70.8% [20]. Comparatively, it is also higher than the rate attained in the East Africa DAPD project I in 2013, where 8.5% of 569 dentists responded [21]. The response to the online questionnaires in this study depicts a tremendous increase. In this study, there were more males than females, which correlates with the sex distribution of dentists in the country, as reported in 2019 [22]. This contrasts with findings in a meta-analysis where females show a higher response rate in the online environment [23]. The majority of the dentists worked in counties housing the four large cities of Kenya. The higher density of dentists in cities in this study corroborates the current distribution of the dental workforce; a disparity in the geographical distribution exists [22]. Nonetheless, the results cannot be generalized to the nation´s 47 counties.

The study's finding that the majority of dentists hold only a Bachelor of Dental Surgery (BDS) qualification aligns with the broader national context in which access to postgraduate training remains limited, particularly in the devolved structure of government. Additionally, the finding that most dentists obtained their primary dental qualifications in Kenya underscores the pivotal role of the two dental schools in shaping the clinical competencies, treatment philosophies, and decision-making approaches of practitioners within the country. These have implications for the quality and scope of oral healthcare services offered at the primary and secondary levels.

Overall, the dentists were knowledgeable about the MCM and DAPD. These results differ from studies among Nigerian and Jordanian dentists who had poor knowledge [2,24]. Similarly, a recent study among UK dentists reported limited knowledge in DAPD among primary care clinicians [25]. The varying levels of knowledge could be attributed to the varying timings of the surveys with reference to the signing of the convention, and or apathy of the matter as reported among Australian dentists in 2014 [26]. It is also probable that the country´s participation in the East Africa DAPD project in 2013 sparked interest among dentists at the inception of the MCM [21]. Additionally, the growing internet penetration has provided avenues to professionals for learning platforms.

A sizeable number of dentists selected composite resins for posterior restoration in permanent dentition, while the use of dental amalgam has reduced considerably. Furthermore, a recent study reported support for the use of mercury-free alternatives among dental interns and final year dental students in Kenya [6]. This contrasts with findings of previous studies done in Kenya in 2009, 2016, where usage of DA was reported as at 50% and 49% respectively [13,27]. Additionally, low usage of composite resins in posterior restorations was reported to range from 25.2% in class I to 18.5% in class II among dentists in Kenya [13]. In particular, the East Africa Dental amalgam phase-down project of 2013 reported equitable high usage of both composite resins and dental amalgam by the majority of the dentists [21]. These findings denote that dental amalgam phase-down is at an advanced level in Kenya. Hence, the current finding joins the global trend in operative practice where virtually all countries have embraced DAPD [3,7,10,28].

In restoring large cavities, a majority select composite resins, whereas half would not select gold and ceramic inlays despite being knowledgeable in DAPD. This relates negatively to preferred management techniques in contemporary operative practice. Use of composite resins in very large cavities has been thought to account for the shift of composite resin restoration failure causes, from secondary caries and wear to restoration fractures and endodontic treatment [15]. While indirect restorations, namely ceramic inlays and gold inlays, are accepted medium-invasive restorations in the absence of DA [29,30]. However, these indirect restorations require intensive capital investment and high-skilled technical support, which is scarce in Kenya and other low and middle-income countries. It is therefore comprehensible that a majority of dentists would select composite resins to perform beyond their indications due to prevailing socio-economic capacities.

Almost all dentists would prepare a cavity for caries extending to the EDJ and outer third of dentine. As dental amalgam eclipses from the range of posterior restoratives, there is a shift towards a preventative, minimally invasive dentistry which entails caries arrest and delayed intervention employing high fluoride and resin infiltrants [4,5]. It can also be argued that with the level of caries burden and the compliance of the average Kenyan patient to review appointments, dentists would rather restore rather than undergo inevitable future root canal treatment interventions. It behooves the dental fraternity to innovatively educate and motivate the population to have a significant caries reduction and smaller cavities in the future. Currently, a caries-free nation remains the holy grail of dentistry in Kenya and globally. Contrastingly, Norwegian dentists postpone restorative interventions until the lesions have progressed to a deeper level [3]. While the current results are in parallel with studies where dentists operatively intervene at too early a stage of caries, but with a shift of dental material to resin composite [3,4,31].

All dental amalgam alternative restoratives are available in the Kenyan market, which is not astounding, as Kenya has the highest concentration of dental materials suppliers in the East and Central African region. This was revealed in the East Africa dental amalgam phase project one [21]. It can further be speculated that conventional glass ionomer cements, which were the most available to a majority of dentists, may be due to their relative affordability and diverse applications in restorative dentistry. Interestingly, bulk fill resin composites were the next accessible DAAR to the majority of dentists, followed by conventional resin composites. Bulk fill resin composites offer ease of manipulation compared to conventional resin composites and have become popular among dentists worldwide [32-34]. Though novel, their clinical performance is similar to that of the long-serving conventional resin composites except for their microhardness and elastic modulus [32,33]. It is not clear why, though knowledgeable in DAPD, the dentists would never select indirect restorative materials to replace DA; similarly, a reluctance to execute minimal intervention to manage early caries.

**Limitations and strengths:** almost half of the dentists were practicing in the four cities in Kenya, hence the results cannot be extrapolated to the 47 counties. However, it may depict the situation in the country, considering that the majority of dentists are in urban centers in Kenya. The study was conducted online, which may have excluded some dentists in some counties internet penetration.

## Conclusion

Kenyan dentists were knowledgeable on the MCM and DAPD; however, more than half were not aware of Kenya´s status with regard to the convention as of 2022. A majority select composite resins to restore very large cavities, and half would never select gold alloys. Almost all dentists would not practice preventive minimally invasive dentistry. The most accessible mercury-free restorative was conventional glass ionomer cement, while the least available was alkasite restorative material. National DAPD guidelines should incorporate capacity building for dentists to integrate dental caries prevention into restorative practice. Additionally, they should promote the use of novel alternatives to dental amalgam, such as alkasite and glass hybrids, and emphasize the appropriate selection of restorative materials for the management of grossly carious teeth.

### What is known about this topic

- The Minamata Convention on Mercury calls for a voluntary phase-down in the use of dental amalgam by countries, and it has enjoined the existing patients' demand for aesthetic restoration, leading to a reduction in the use of dental amalgam in posterior teeth worldwide;
- Dentists are key stakeholders in DAPD; their knowledge of the MCM and DAPD and uptake of use of alternative restoratives in molars and premolars has culminated in a paradigm shift in operative dentistry;
- Dental curriculum change for dental students and continuous professional development for dentists has been embraced to prepare the dental workforce to transition from the mainstay use of dental amalgam to a preventative, minimally invasive dentistry philosophy enshrined in the dental amalgam phase down.

### What this study adds

- The study revealed that Kenyan dentists are knowledgeable about the global Minamata Convention on Mercury and the phase-down of dental amalgam, but they have not followed the ratification process of the convention in the country;
- The study provides evidence that Kenya is at an advanced stage in phasing down the use of dental amalgam, with most dentists utilizing resin composite as an alternative, which aligns with trends in restorative practice globally;
- Kenyan dentists have yet to adopt minimally invasive dentistry and continue to favor the traditional restorative model, underscoring the need for enhanced training in preventive care.
